# Cytoarchitectonic Areas of the *Gyrus ambiens* in the Human Brain

**DOI:** 10.3389/fnana.2019.00021

**Published:** 2019-02-21

**Authors:** Ricardo Insausti, Marta Córcoles-Parada, Mar Maria Ubero, Adriana Rodado, Ana Maria Insausti, Mónica Muñoz-López

**Affiliations:** ^1^Human Neuroanatomy Laboratory, School of Medicine, University of Castilla–La Mancha, Albacete, Spain; ^2^Departamento de Anatomía, Universidad Católica San Antonio de Murcia, Murcia, Spain; ^3^Faculty of Health Sciences, University Public of Navarra, Pamplona, Spain

**Keywords:** human, entorhinal cortex, subfield EMI, ambient gyrus, cytoarchitectonics, BA34

## Abstract

The *Gyrus ambiens* is a gross anatomical prominence in the medial temporal lobe (MTL), associated closely with Brodmann area 34 (BA34). It is formed largely by the medial intermediate subfield of the entorhinal cortex (EC) [Brodmann area 28 (BA28)]. Although the MTL has been widely studied due to its well-known role on memory and spatial information, the anatomical relationship between *G. ambiens*, BA34, and medial intermediate EC subfield has not been completely defined, in particular whether BA34 is part of the EC or a different type of cortex. In order to clarify this issue, we carried out a detailed analysis of 37 human MTLs, determining the exact location of medial intermediate EC subfield and its extent within the *G. ambiens*, its cortical thickness, and the histological–MRI correspondence of the *G. ambiens* with the medial intermediate EC subfield in 10 *ex vivo* MRI. Our results show that the *G. ambiens* is limited between two small sulci in the medial aspect of the MTL, which correspond almost perfectly to the extent of the medial intermediate EC subfield, although the rostral and caudal extensions of the *G. ambiens* may extend to the olfactory (rostrally) and intermediate (caudally) entorhinal subfields. Moreover, the cortical thickness averaged 2.5 mm (1.3 mm for layers I–III and 1 mm for layers V–VI). Moreover, distance among different landmarks visible in the MRI scans which are relevant to the identification of the *G. ambiens* in MRI are provided. These results suggest that BA34 is a part of the EC that fits best with the medial intermediate subfield. The histological data, together with the *ex vivo* MRI identification and thickness of these structures may be of use when assessing changes in MRI scans in clinical settings, such as Alzheimer disease.

## Introduction

The entorhinal cortex (EC) or *Cortex Entorhinalis*^1^ is a component of the hippocampal formation (HF), which is formed by different archicortical (dentate gyrus, CA fields, subiculum) and periarchicortical areas (presubiculum, parasubiculum, EC). In humans, the EC forms part of the parahippocampal gyrus (PHG) or *Gyrus parahippocampalis.* The PHG is located in the ventromedial surface of the temporal lobe, which, with the adjacent cortex lining the collateral sulcus *or sulcus collateralis*, forms the medial temporal lobe (MTL).

The MTL has been associated to functions such as memory and processing of spatial information (Aggleton and Mishkin, 1985; Maguire et al., 2000; Squire et al., 2004). The vast majority of the information entering the nonhuman primate EC comes exclusively from polysensory association areas (for review, see Insausti et al., 2017). The majority of the cortical input reaches the upper layers of EC subfields over the rostro-caudal and medio-lateral extents of the EC (Suzuki and Amaral, 1994; Insausti and Amaral, 2008; Mohedano-Moriano et al., 2008). The cortical output leaves the EC through the deep layers (V and VI) and sends information back to polysensory areas that send input to EC (Muñoz and Insausti, 2005). Pathological processes in the EC are considered as one of the hallmarks of Alzheimer disease (Braak and Braak, 1992) with a decrease in neuron number occurs, not only in upper layers (Gómez-Isla et al., 1996), but also in deep layers as well (Arnold et al., 1991).

Along the years, the study of the MTL, and in particular in the field of Alzheimer disease, has also incorporated neuroimaging techniques which can determine both the location of the EC in MRI images (Insausti et al., 1998a), as well as its structural and volumetric changes (Juottonen et al., 1998; Khan et al., 2014).

The EC laminar structure is known since Hammarberg (1895) and can be found in classical neuroanatomical work (Cajal, 1901; Lorente de Nó, 1933), although it was not topographically described until Brodmann (1909). He divided the MTL into area 28 (EC), located in the anterior portion of the PHG, and area 34, which lies medial to area 28. BA34 corresponds approximately to the macroscopically visible ambient gyrus or *Gyrus ambiens (GA)*. The GA forms in the human brain a visible protuberance situated in the medialmost portion of the anterior temporal lobe. This parcellation of the cerebral cortex is not completely accepted by contemporary authors who challenge the correspondence of Brodmann’s cortical areas as defined by functional studies (Nieuwenhuys et al., 2015).

The GA as an anatomical structure is known since Solly (1836), who described it in relation to the *limen insulae* (Swanson, 2015). The GA was assigned first to the olfactory system and later included as part of the piriform area by Smith in 1919 (Swanson, 2015). Williams and Warwick (1980) include the GA as part of the prepiriform area, which continues caudally into the entorhinal area.

**FIGURE 1:**
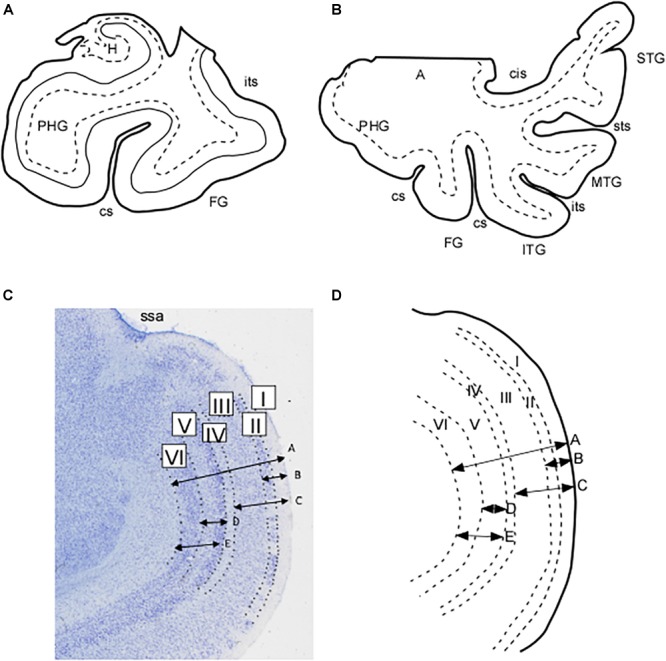
Schematic representation of the extent of the blocks employed in this study. **(A)** Block limited to the parahippocampal and fusiform gyri. **(B)** Blocks of the whole temporal lobe as far as the circular sulcus of the insula. **(C)** Microphotograph in which the layers of the EC subfield E_MI_ can be appreciated. Arrows represent the measurements made of the total and partial thicknesses of the cortex in E_MI_. These are represented schematically in **(D)** as indicated: A, total thickness of subfield E_MI_; B, thickness of layers I and II; C, thickness of layers I–III; D, thickness of layer V; E, thickness of layers V and VI. Abbreviations: A, amygdala; cs, collateral sulcus; FG, fusiform gyrus; H, hippocampus; ITG, inferior temporal gyrus; its, inferior temporal sulcus; MTG, middle temporal gyrus; PHG, parahippocampal gyrus; ssa, *sulcus semianularis*; STG, superior temporal gyrus; sts, superior temporal sulcus.

The GA (Figure 1A,B) is the largest and most outstanding gross macroscopic prominence of the PHG. Other bulge in the most anterior part of the MTL is the semilunar gyrus (GS), separated from the GA by the semianular sulcus or *sulcus semianularis*^2^. Cytoarchitectonically, the GS corresponds to periamygdaloid cortex (Insausti and Amaral, 2012). Another bulge close to the GA is uncinate gyrus (GU), which lies caudal to the GA and GS, at the transition between the caudalmost portion of the amygdala and the hippocampus (Figure 1B). Therefore, the GA can be considered as a medial extension of the PHG in the MTL.

As mentioned above, the cortex situated in the anterior part of the PHG is the EC, a phylogenetically conserved brain region present in all mammals (Stephan, 1975). The cytoarchitecture of the EC has been subject of study since the middle of the nineteenth century, and it has been subdivided into different subfields (Amaral et al., 1987; Insausti et al., 1995, and references therein). The best-known synonym of EC is BA28, and both are used in an interchangeable way. Somewhat less clear is the type of cortex present in BA34. Brodmann describes it as: “*Area 34 – the dorsal entorhinal area*. *It lies mainly to the inferior rhinencephalic sulcus* (*Retzius, 1896*), *so that this sulcus forms the approximate border between both types*” (Brodmann, 1909, translation of Garey, 1994). The other area alluded to is BA28 or EC. It is obvious that Brodmann only gives an account of the location of his dorsal entorhinal area, without any further elaboration on cytoarchitectonic characteristics.

Brodmann’s gross anatomical drawing of the human cortical area shows that area 34 is largely coincident with the macroscopic GA. Moreover, Brodmann indicates the “inferior rhinencephalic sulcus” (Retzius, 1896) as the boundary between areas 28 and 34 and refers to the cortex of the “lunate gyrus” of Retzius (equivalent to GS) on the medial side of area 34 (Figure 1A). Therefore, Brodmann makes a clear distinction of two separate areas, area 28 or EC, and area 34 in the anterior part of the PHG. Area 34 is still in use by some authors (Krimer et al., 1997; Ding, 2013).

The gross anatomical organization of the MTL has been dealt with in several reports (Amaral and Insausti, 1990; Duvernoy, 2005; Insausti and Amaral, 2012), and they all agree with the commonly accepted pattern of the series of bulges present in the medialmost aspect of the MTL. However, the pattern of sulci present in the PHG is more controversial, and reports in the literature show a disparity of names (Heckers et al., 1990; Ono et al., 1990; Heinsen et al., 1996; Hanke, 1997; Duvernoy, 2005; Huntgeburth and Petrides, 2012). Finally, although the cytoarchitectonic segmentation of the human EC has been studied along the last century, some differences still persist (Insausti et al., 1995; Krimer et al., 1997).

Modern MRI techniques reveal the anatomy of the MTL in detail and great accuracy (Yushkevich et al., 2015; Iglesias et al., 2016), so that the shape and extension of the medial temporal bulges (GA, GS, and GU) can be readily assessed. Thereby, the anatomical identification of the medial temporal prominences (Stephan, 1975; Amaral and Insausti, 1990; Duvernoy, 2005; Insausti and Amaral, 2012; Ding and Van Hoesen, 2015) makes it possible to use its morphometry in studies on neurodegenerative diseases, such as in Alzheimer disease.

The aim of this descriptive study is twofold. First, we sought to explore the detailed macroscopic anatomy of the GA, and its relationship with other bulges and sulci at the upper MTL, in order to check the feasibility of MRI identification of the GA in relation to other medial temporal landmarks. Second, we wanted to determine the cytoarchitectonic fields that conform the GA, both at the mediolateral and rostrocaudal extents, as our previous cytoarchitectonic parcellation of the human EC suggest that the GA may contain more than one EC subfield (Insausti et al., 1995).

## Materials and Methods

The study was performed according to the Declaration of Helsinki and approved by the Ethical Committee on Clinical Research of the University Hospital of Albacete (meeting of January 2015).

The results presented are based on observations of 37 MTLs of control cases, ranging between 12 and 110 years, from the Human Neuroanatomy Laboratory archive at the University of Navarra (1985–1998) and the University of Castilla–La Mancha (1999–present). Most of them were fixed by immersion in 10% buffered formalin for at least 4 weeks. Brains were photographed and blocked in 1 cm thick slabs (coronal plane), either perpendicular to the line traced along the anterior and posterior commissures as previously used in other studies (Insausti et al., 1995), or taking the posterior end of both mammillary bodies as references.

**FIGURE 2:**
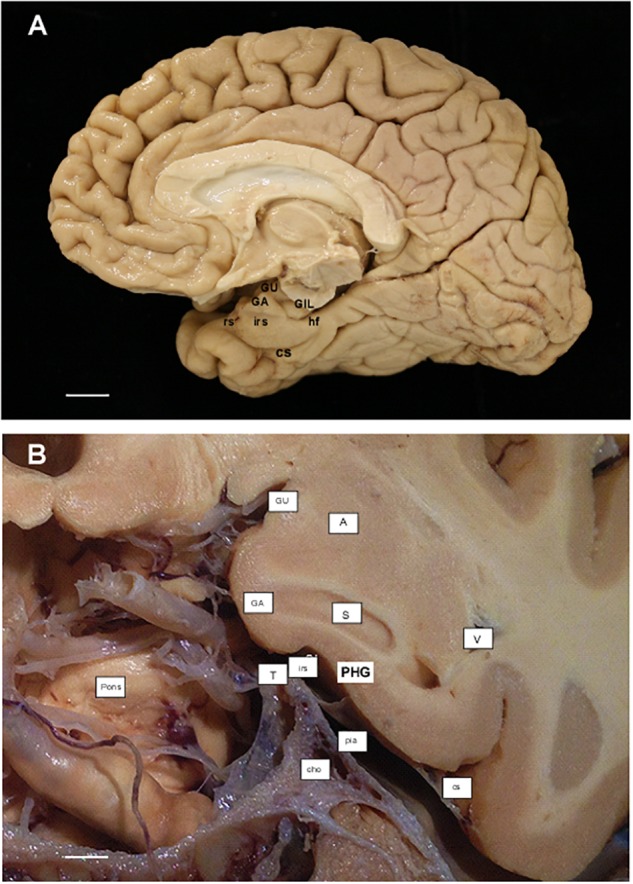
**(A)** Photograph of the medial aspect of a cerebral hemisphere showing the main gross anatomical structures in relation to the remainder of cerebral structures. Scale bar equals 1 cm. **(B)** Photograph of a cadaveric preparation of a coronal section of the head at the level of the Subiculum (start of the hippocampus). Notice the ventral limit of the intrarhinal sulcus in relation to the *G. ambiens* (GA). The free edge of the tentorium (T) can be appreciated in close proximity to the intrarhinal sulcus. Scale bar equals 5 mm. Abbreviations as in previous Figure 1: cho, anterior choroidal artery; GA, *Gyrus ambiens*; GIL, *Gyrus intralimbicus*; GU, *Gyrus uncinatus*; hf, hippocampal fissure; irs, intrarhinal sulcus; pia, pia mater; Pons, anterior aspect of the pons; rs, rhinal sulcus; S, subiculum; T, tentorium; V, lateral ventricle, temporal horn.

The anterior and posterior surfaces of the slabs were individually photographed and the MTL was dissected through a medial cut along the entorhinal sulcus (Gloor, 1997) or posterior to the end of the *Gyrus intralimbicus*, at the most caudal level of the uncus, also known as the uncal apex by Duvernoy (2005), along the choroidal fissure (*fissura choroidea*). The block of tissue encompassed either the lateral occipitotemporal sulcus (*sulcus occipitotemporalis lateralis*) as presented in Figure 2A, or the most complete temporal lobe by the circular sulcus of the insula (*sulcus circularis insulae*) at the level of the insular cortex (Figure 2B). The dissected temporal lobe blocks included from the temporal pole and anterior PHG to the end of the hippocampus and associated cortices at the beginning of the lingual gyrus (*Gyrus lingualis*).

The dissected blocks were placed in 4% paraformaldehyde in 0.1 M phosphate buffer for a period of 2–4 weeks. Serial 50 μm thickness sections were obtained with the use of a sliding microtome coupled to a freezing unit as described previously (Insausti et al., 1995; Blaizot et al., 2010). One-in-ten sections were Nissl stained with 0.25% thionin for cytoarchitectonic evaluation (500 μm interval between adjacent sections).

Annotation of boundaries was made by means of a *camera lucida* attached to a Nikon SMZ or a Leica MZ6 stereomicroscope. In all cases, a thorough analysis of the cytoarchitectonic features was made on the basis of previously reported criteria for the EC (Insausti et al., 1995), perirhinal cortex^3^ (Salinas, 1995), and posterior parahippocampal cortex^4^ (Insausti, 1992). Sections throughout the GA were selected for estimation of total thickness, as well as that of its layers (Figure 2).

In each case the linear distance between the pia mater and the boundary with the white matter was measured, along with different combinations of layers and cell free spaces up to five length determinations: (1) total thickness of EC subfield E_MI_ taken at the most convex point of the GA; (2) thickness of layer I plus layer II, which estimates the value of the layer origin of the projection to the molecular layer of the dentate gyrus; (3) thickness of the sum of layers I, II, and III as an estimate of the layers that project both to the CA fields of the hippocampus and to the dentate gyrus; (4) layer V thickness as an account of the main output layer of the EC; (5) combined thickness of layers V and VI as a more complete EC output. Figure 1C,D show a microphotograph of the GA with representation of the distances, as well as its schematic representation. The results for the distances are presented in Table 1.

**Table 1:** Values of the thickness of the EC subfield E_MI_.

Cases	Age	Layers				
		I–VI	I–II	I–III	V	V–VI
Case 1	71	2.8	0.7	1.5	0.3	1.2
Case 2	58	2.3	0.5	1.2	0.4	0.8
Case 3	78	3.2	0.4	1.3	0.3	1.2
Case 4	82	2.0	0.6	0.8	0.3	0.8
Case 5	83	3.0	0.5	1.5	0.4	0.9
Case 6	59	2.6	0.5	1.2	0.5	1.1
Case 7	90	3.0	0.6	1.5	0.5	1.2
Case 8	61	2.5	0.6	1.3	0.5	1.0
Case 9	83	2.3	0.5	1.3	0.4	0.9
Case 10	75	2.2	0.4	1.2	0.3	0.8
	Mean	2.59	0.52	1.30	0.40	1.00
	*SD*	0.39	0.87	0.30	0.09	0.15

In order to complete the measurement of the GA, two-dimensional maps were constructed and measured with a planimetric program as described previously (Insausti et al., 1998b). In this study, the unfolding corresponds to the middle of the EC, either through *lamina dissecans* or the interval between layers III and V when the *lamina dissecans* is absent.

Finally, a total of 10 control subjects MRI from the Radiology Service of the Albacete University Hospital were assessed. Distances from the temporal pole to the *limen insulae*, the beginning of the hippocampus (subiculum), and the distance between the intrarhinal sulcus and the *Gyrus intralimbicus* was also calculated as an indirect estimation of the GA extent, calculated from the difference of the two distances alluded to above. The radiological parameters have been reported previously (Delgado-Gonzalez et al., 2015).

## Results

### Extent and Limits of the *Gyrus ambiens*

The term GA may be derived from its positions facing the ambient cistern (*cisterna ambiens*), as a descriptive term (latin, *ambiens*, something that surrounds) that is one of the cerebrospinal fluid spaces in the brain, surrounding the upper part of the mesencephalon, vessels, and nerves (Figure 1A,B).

In our series, the GA is present in all cases medial to the PHG. Two sulci, one dorsal and one ventral, demarcate the GA in the upper, medial part of the MTL. The sulcus located more dorsally is the *sulcus semianularis* (Figure 1A,B). This sulcus is located at the rostromedial part of the external surface of the amygdaloid complex [cortical part, which is considered as peripaleocortex (Stephan and Andy, 1970)]. Dorsal to the *sulcus semianularis* lies the periamygdaloid cortex and other medial amygdaloid nuclei; ventral to it lies the GA, so that it can be considered as the boundary between the GA and periamygdaloid cortex. This was a constant feature in our series, although in some cases the periamygdaloid cortex straddled over the *sulcus semianularis* for a few hundred microns.

The GA is limited ventrally by the intrarhinal sulcus in all our cases (Figure 1B), although in some cases it was very shallow and inconspicuous. This small sulcus runs approximately in the most medial one-third of the distance between the *sulcus semianularis* and the collateral sulcus. The GA is coincident with the point at which, in a series of coronal sections, the PHG reaches its maximal breadth. At this midpoint of the PHG, the medial shoulder of the collateral sulcus forms the ventral limit of the PHG, while its lateral bank and shoulder belongs to the fusiform gyrus (*Gyrus fusiformis*), also known as lateral occipitotemporal gyrus (*Gyrus occipitotemporalis lateralis*) (Nieuwenhuys et al., 2008).

In our series of temporal lobes, the intrarhinal sulcus starts at approximately 9 mm behind the frontotemporal junction (*limen insulae*). At this point, the GA begins to be visible on the anterior part of the PHG. The intrarhinal sulcus extends longitudinally for several millimeters (7–10 mm), although we have observed its shape to vary from a small dimple to a marked depression. Sometimes, a secondary additional sulcus could be appreciated on the surface of the PHG. Moreover, in our series of coronal sections, the end of the intrarhinal sulcus coincided with the start of the hippocampal fissure (*fissura hippocampalis*) (Amaral and Insausti, 1990; Insausti and Amaral, 2012). In most cases, the intrarhinal sulcus extends as far back as the GA, which corresponds to the level of the hippocampal head. A great symmetry in length of the intrarhinal sulcus between left and right hemispheres was found in our sample of MRI cases (*n* = 10)^5^. Dorsal to the GA, the GS continues with the GU, which corresponds to the amygdalo-hippocampal area and ends as the hippocampal amygdaloid transitional area (HATA of Rosene and Van Hoesen, 1987; Ding and Van Hoesen, 2015).

The pattern of appearance of the intrarhinal sulcus and the GA above described is the most common in our series of brains, regardless of age. Other variations in the morphology of the GA depend on the presence of other smaller indentations at the surface of the PHG.

### Cytoarchitectonics of the *Gyrus ambiens* Cortex

We present the topological and cytoarchitectonic features of the cortex of the GA, and its relationship with the cortex of the remainder of the PHG at this level.

Most of the PHG cortex is occupied by an intervening type of cortex, the periallocortex, located between the allocortical fields of the hippocampus, subiculum (three layers), and proisocortex (six layers); the latter also known as mesocortex in the current neuroanatomical nomenclature (cortex of five or six layers, with incomplete lamination scheme). It is the EC, along with the presubiculum and parasubiculum, that form the periallocortex of the PHG (Braak, 1980; Insausti et al., 2017). The basic laminar structure of the EC is of six layers and, although they are also named as layers I–VI, they do not correspond to the layers found in the isocortex (neocortex). The EC presents gradual variation along the anterior–posterior and medial–lateral axes, which justifies the separation of up to eight subfields, in which the transition of one EC subfield to another is gradual rather than sharp (Insausti et al., 1995).

**FIGURE 3:**
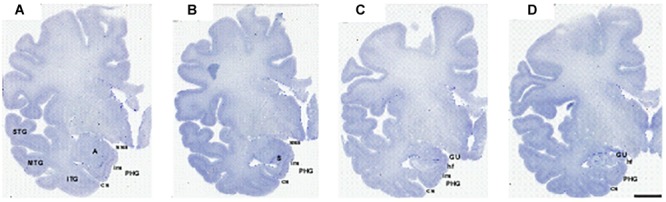
Series of photomicrographs of a whole cerebral hemisphere in coronal sections from rostral **(A)** to caudal **(D)**, in which the relationship of the GA with the hemisphere and the intrarhinal sulcus are displayed. In panel **(A)**, the rostral level of GA is at the level of the mid amygdala. Panel **(B)** shows that the maximal extent of the GA is at the level of the commencement of the Subiculum (S). Panel **(C)** is at the end of the intrarhinal sulcus at the transition with the GU, near the opening of the hippocampal fissure. Note the *sulcus semianularis* is still evident in panels **(A–C)**. Panel **(D)** shows the GU past the intrarhinal sulcus at the level of the hippocampus–amygdaloid transitional area (HATA). Abbreviations as in previous figures. Scale bar equals 1 cm.

**FIGURE 4:**
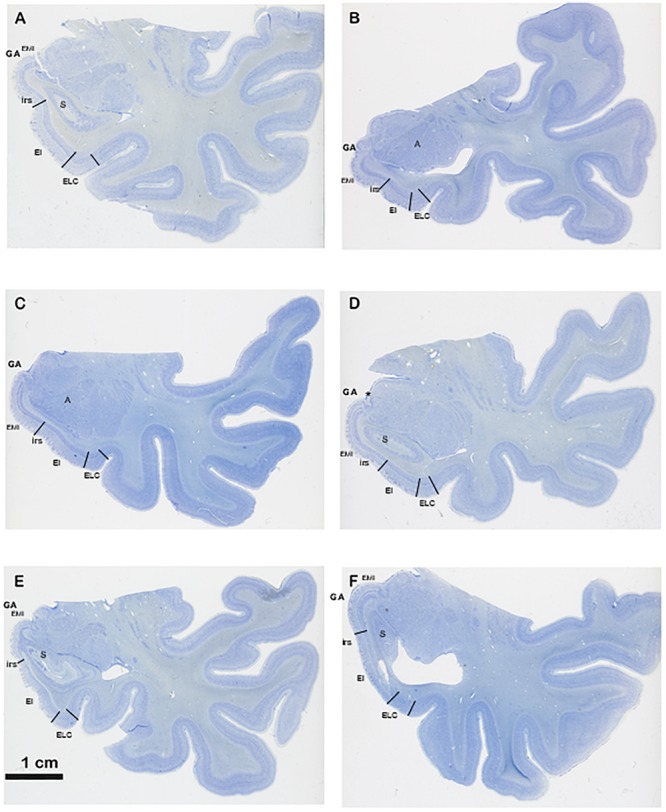
Series of six different cases at the level of the commencement of the Subiculum. The series show different shapes and morphological variability of the GA, in particular differences in depth of the intrarhinal sulcus. Note the sequence from deeper intrarhinal sulcus **(A–C)** to shallower **(D,E)**, to very shallow **(F)**. Abbreviations as in previous figures: EMI, medial intermediate subfield of the EC; EI, intermediate subfield of the EC; ELC, lateral caudal subfield of the EC. Scala bar equals 1 cm.

According to the lamination scheme in terms of number and organization of the cortical layers, we determined in our series that the GA, like the EC, is made up of periallocortex. Besides, the continuation between the GA cortex and the remainder of EC is very noticeable (Figure 3, 4). Rostromedially, the GS cortex can be considered as peripaleocortex; it occupies the extensive region that borders the boundary between the periallocortex of the GA and the peripaleocortex of the GS. In a caudal direction, and once the hippocampal fissure is present, the GA comes to an end, and its location is now taken by the GU, which corresponds to the amygdalo hippocampal area, and further caudally by the hippocampus amygdaloid transitional area (Rosene and Van Hoesen, 1987). Therefore, the GA is limited dorsally by the GS, ventrally by the periallocortex of the EC, rostrally by the anterior portion of the EC, and caudally by the GU. Two of the boundaries (ventral and rostral) correspond to EC while the other two (dorsal and caudal) correspond to parts of the amygdaloid complex.

Our observations indicate that the GA consists of periallocortex, similar to the EC in number and organization of the layers. The main topological features of EC subfields rostrally adjacent to the GA are as follows, according to the nomenclature of Insausti et al. (1995). The anterior pole of the EC shows subfields Entorhinal Olfactory subfield (E_O_) and Entorhinal Lateral Rostral subfield (E_LR_), which are the most rostrally located subfields. E_O_ resembles the periamygdaloid cortex in terms of appearance (both present a conspicuous layer II organized in clumps) although subfield E_O_ shows six layers, as the remainder of the EC, in contrast to the three layers of periamygdaloid cortex. Subfield E_O_ abuts laterally subfield E_LR_ (for details, see Insausti et al., 1995). Immediately caudal to E_O_ subfield is Entorhinal Rostral subfield (E_R_), which also borders the GA anteriorly. Subfield E_R_ presents six layers completely developed, although it lacks *lamina dissecans*. In a caudal direction the dorsomedial aspect of the EC corresponds to Entorhinal Medial Intermediate subfield (E_MI_), which is the periallocortex that, along with the incipient angular bundle, makes up the GA.

E_MI_ is very noticeable because of specific features. First, E_MI_ shows clearly the typical and complete set of layers that characterize the EC. Interestingly, this subfield resembles closely the appearance of the monkey EC, especially at midlevel. Second, it can be accurately distinguished in coronal sections as the medial prominence formed by the GA. In this way, subfield E_MI_ covers the majority of the macroscopically GA (Figure 3, 4). In most cases, it is separated from the most ventral part of the periamygdaloid cortex by a conspicuous cell-free space in the medial border of E_MI_, which lies slightly ventral to the *sulcus semianularis* (Figure 4D). The remainder of the EC is in the macroscopically visible PHG. The common features of EC layering are present in all fields, but it is subfield E_MI_ that displays them more clearly. Figure 1 shows plainly the stack of layers that characterizes subfield E_MI_. Although the cytoarchitectural features of subfield E_MI_ have been reported previously (Insausti et al., 1995), briefly, layer I is made up of fibers in continuation with the layers in the periamygdaloid cortex. The pial surface looks smoother relative to other portions of the EC, and, although subfield E_MI_ also presents *verrucae hippocampi*^6^ their appearance is somewhat flatter. Layer II is narrow and more akin to layer II of subfield E_O_. A thin cell-poor stratum interposes between layers II and III. Layer III is made up of small pyramids, orderly arranged in unicellular columns. A drop in the density in the deep portion of layer III announces *lamina dissecans*. Layer IV is *lamina dissecans*, one of the most outstanding features of the human periallocortical regions (Insausti et al., 2017). *Lamina dissecans* presents a cell-free space, which extends from the border with layer III to the big pyramids that populate the upper portion of layer V. Layer V is made up of three sublayers: Va, which contains densely packed big and dark pyramids; Vb, which displays lower density of pyramids, otherwise similar to those in sublayer Va; and Vc, a cell-poor stratum which runs parallel to *lamina dissecans*. Layer VI is also multilayered, although not as clearly as layer V. For this reason, this is the only subfield that displays clearly two cell-free bands, *lamina dissecans* and sublayer Vc, on either side of the dark, big pyramids of layer V, parallel to the convex surface of the GA. While subfield E_I_ also presents *lamina dissecans* and sublayer Vc, it displays a less distinct appearance relative to E_MI_. Likewise, subfields E_C_ and E_CL_, located caudally to E_I_, also show a prominent sublayer Vc, although they do not display *lamina dissecans* (Insausti et al., 1995).

For all of the above, periallocortex of the GA is the same as the cytoarchitectonic type of subfield E_MI_, and thence, EC as one of its subfields (Insausti et al., 1995). There is no other periallocortical field between the GA and the amygdaloid complex, and thereby we must conclude that BA34 is the same as EC subfield E_MI_ as defined here, and therefore, a part of BA28, not a different one.

**FIGURE 5:**
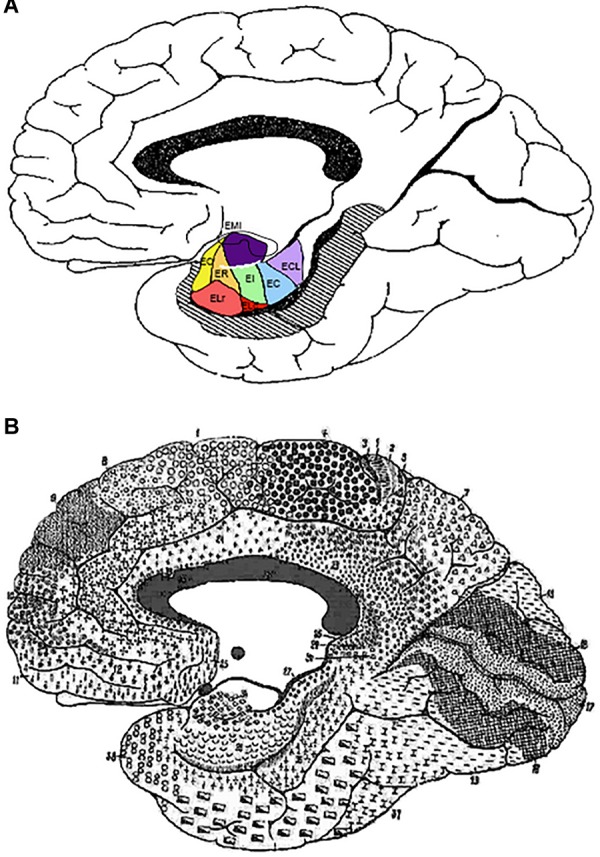
**(A)** Depiction of the outline of Brodmann’s map of the medial surface of the brain. It shows the approximate transformation of EC subfields according to the parcellation of Insausti et al. (1995) into Brodmann’s scheme. **(B)** Brodmann (1909) cytoarchitectonic map. Note the overall correspondence of EC subfield E_MI_ with BA34. Abbreviations as in previous figures: EC, caudal subfield of the EC; ECL, caudal limiting subfield of the EC; ELr, lateral rostral subfield of the EC; EO, olfactory subfield of the EC; ER, rostral subfield of the EC.

In conclusion, the continuity of the EC along the extent of the GA is given by (a) the ventral continuation of E_MI_ cytoarchitectonic layers with the subfield E_I_ in continuation of the EC; (b) the structural difference with the medially adjacent periamygdaloid cortex (peripaleocortex). The rostral part of E_MI_ may encroach upon E_O_ and E_R_, and that completes the GA area. The GA extension is completed by the rostral part of E_O_ and E_R_, as shown in Figure 5.

### Morphometric Parameters of EntorhinalCortex Subfield E_MI_

#### Cortical Thickness of GA/E_MI_

In an anterior to posterior direction, the longitudinal extent of the EC is about 2.5 cm. The GA lies at the middle of the EC (Figure 2, 5). We completed this longitudinal distance with the measurement of the EC subfield E_MI_ in Table 1, corresponding to 10 control cases (Age range, 58–90).

The total thickness of EC subfield EMI ranged between 2 and 3.2 mm [mean 2.59; standard deviation (SD) ± 0.39], and it is relatively constant regardless of the age of the subject. Values for layers I–II of the subfield EMI range from 0.4 to 0.7 mm (mean 0.52; *SD*±0.87). Layers I–III (EMI upper layers) resulted in values that ranged between 0.8 and 1.5 mm (mean 1.30; *SD* ±0.30). Layer V thickness was the smallest value, as it corresponds to a single layer; values ranged between 0.3 and 0.5 mm (mean 0.40; *SD* ±0.09). The addition of layers V and VI varied between 0.8 and 1.2 mm (mean 1.00; *SD* ±0.15). Data are presented in Table 1, and show that the range of variability is low, probably due to the sharpness of the limits among layers in this EC subfield.

#### Two-Dimensional Reconstruction Measurementsof E_MI_

Two-dimensional reconstructions allow the measurement of the extent of any cortical area. EC has been unfolded taking the middle of the EC thickness as the unfolding line. Subfield E_MI_ extent was unfolded, and the values expressed as percentage of the total EC unfolded surface. Data from a representative number of cases are shown in Table 2. Age ranged from 15 to 110 years to offer a glimpse of the complete lifespan, excluding childhood. All measurements are expressed in mm^2^ as absolute value, also the value of E_MI_ extent, which is also shown as a percentage of the total EC surface.

**Table 2:** Values of the areal extent of subfield E_MI_.

Cases	Age	EC (mm^2^)	E_MI_ (mm^2^)	%
Case 1	15	244.7	14.5	5.9
Case 2	22	213.5	12.0	5.6
Case 3	32	312.8	13.0	4.2
Case 4	54	297.5	5.0	1.7
Case 5	54	350.7	15.0	4.3
Case 6	54	345.8	9.5	2.7
Case 7	58	331.9	8.0	2.4
Case 8	61	340.5	6.5	1.9
Case 9	62	207.2	8.5	4.1
Case 10	63	260.0	12.5	4.8
Case 11	64	315.8	7.0	2.2
Case 12	64	302.6	6.0	2.0
Case 13	66	336.7	12.5	3.7
Case 14	70	341.9	5.0	1.5
Case 15	71	254.2	13.5	5.3
Case 16	77	268.6	6.5	2.4
Case 17	77	256.4	8.0	3.1
Case 18	78	351.1	7.5	2.1
Case 19	83	197.6	12.5	6.3
Case 20	84	335.8	6.0	1.8
Case 21	84	217.2	5.0	2.3
Case 22	84	309.6	9.5	3.1
Case 23	85	211.9	5.5	2.6
Case 24	85	269.2	8.0	3.0
Case 25	87	211.3	5.5	2.6
Case 26	91	197.3	7.0	3.5
Case 27	110	196.0	8.0	4.1
	Mean	277.0	8.8	3.3
	*SD*	56.0	3.2	

The EC total surface ranged from 350 to 197 mm^2^. Values under 200 mm^2^ corresponded to the two oldest representative cases, 91 and 110 years, respectively (197.3 and 196.0 mm^2^). Interestingly, in those two old cases, the values of E_MI_ were in the normal range. Values of E_MI_ extent ranged from 15.0 to 5.0 mm^2^. Therefore, the percentage of the EC total surface ranged between 1.5 and 6.3%. It is noteworthy to point that the oldest case in our series showed a percentage of 4.1%, which is in the high range in percentage of all series.

### MRI Appearance of the *Gyrus ambiens*

The location and extent of the GA in the MTL was established, and the rostrocaudal dimension determined, as well as reference distances with different landmarks used on former MRI studies (Insausti et al., 1998b; Franko et al., 2014).

**FIGURE 6:**
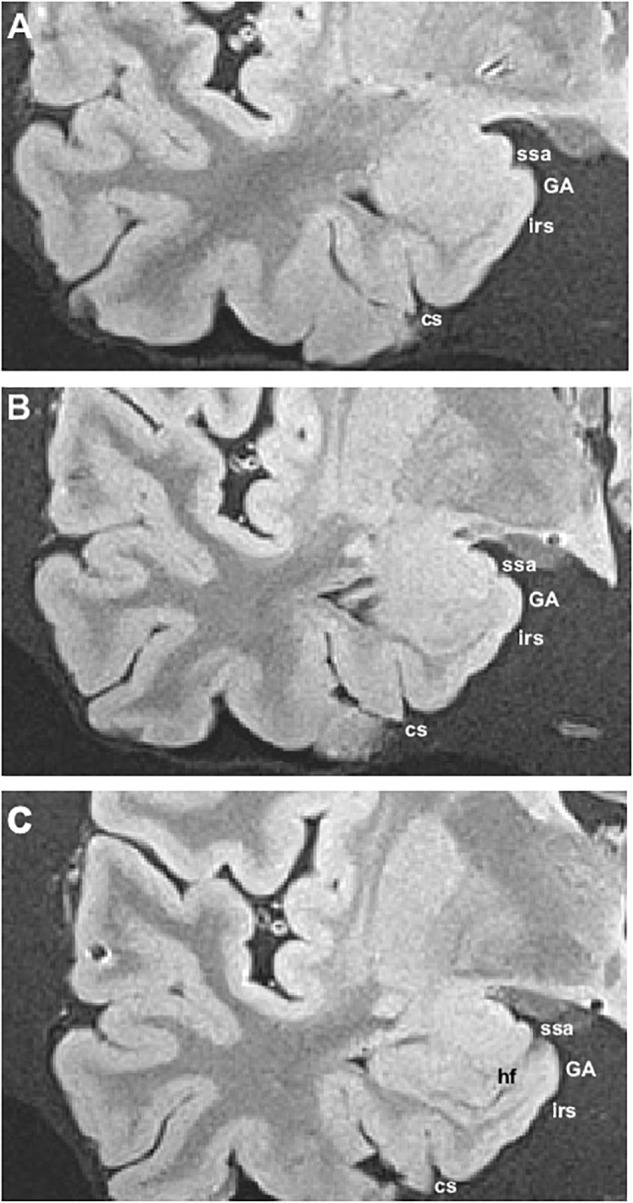
*Ex vivo* MRI appearance of the GA in coronal sections of three different cases. The boundary of the GA (subfield E_MI_) was determined by histological examination. Abbreviations as in previous figures.

The series of coronal sections in MRI included levels at which the GA can be recognized. The main landmark for identification of the GA is at the level where the temporal horn of the lateral ventricle starts, or at the level of the start of the subiculum, slightly caudal to the lateral ventricle starting point. Very often, the *sulcus semianularis* separating the GA and the GS is visible, signaling the dorsal boundary of the GA. The intrarhinal sulcus is also visible in the PHG, although its depth may be somewhat variable. An example of the radiological appearance of the GA is shown in Figure 6.

We determined various parameters in relation to distance from the temporal pole, *limen insulae* (frontotemporal junction), start of the subiculum, and the end of the uncus (*Gyrus intralimbicus*), aiming at establishing the location and rostrocaudal extent of the GA in MRI images of 10 human control cases. Table 3 presents the distances among those landmarks. The distance from the beginning of the temporal pole and the *limen insulae* was fairly constant, 2.5 cm as an average. The EC starts about 2 mm behind the *limen insulae*. The bulge indicating the GA was considered the start of the intrarhinal sulcus. From that point, the distance to the start of the subiculum gave values between 7.2 and 14.4 mm (mean 9.36 mm; *SD* ±2.39 for the left hemisphere and 9.12 mm; *SD* ±2.48 for the right hemisphere). The length between the start of the hippocampal fissure and the end of the hippocampal head in the rostrocaudal axis varied between 21 and 14 mm (left hemisphere, mean 18.96 mm; *SD* ±2.39; right hemisphere, mean 18.48 mm; *SD* ±3.59).

**Table 3:** Length values among MTL landmarks in relation to the GA.

Cases	Temporal pole – *limen insulae*		irs-subiculum		irs-*Gyrus intralimbicus*	
	Left hemisphere	Right hemisphere	Left hemisphere	Right hemisphere	Left hemisphere	Right hemisphere
Case 1	24.0	24.0	9.6	7.2	19.2	19.2
Case 2	26.4	24.0	9.6	9.6	19.2	16.8
Case 3	21.6	24.0	12.0	12.0	21.6	19.2
Case 4	21.6	21.6	7.2	7.2	16.8	14.4
Case 5	24.0	21.6	7.2	9.6	16.8	21.6
Case 6	26.4	26.4	7.2	7.2	16.8	16.8
Case 7	26.4	24.0	9.6	9.6	19.2	19.2
Case 8	24.0	21.6	7.2	7.2	16.8	16.8
Case 9	24.0	24.0	9.6	7.2	19.2	14.4
Case 10	38.4	36.0	14.4	14.4	24.0	26.4
Mean	25.68	24.72	9.36	9.12	18.96	18.48
*SD*	4.81	4.24	2.39	2.48	2.39	3.59

*irs, intrarhinal sulcus.*

## Discussion

### Intrarhinal Sulcus and the GA

In the present report we show that the ventral limit of EC subfield E_MI_ (and therefore of the GA) corresponds topographically to the intrarhinal sulcus. The existence of a sulcus that delineates the ventral limit of the GA is well accepted in the literature, although the terminology employed varies among authors.

The sulci of the MTL surface have been examined by different authors, who have shown the variability of the sulci present in the EC. A detailed study of those EC sulci is reported by Hanke (1997), where he refers as “intrarhinal nick” to the sulcus ventral to the GA. Specifically, he states that “the intrarhinal nick forming the lateral border of the ambient gyrus, corresponds to the impression of the of the anterior petroclinoideal plica of the cerebellar tent, and was lacking in 30.4% of the cases.” This suggests that, at the gross morphological level, the intrarhinal sulcus is present in more than two-thirds of the population, thus making it a rather constant feature of the EC. We noticed in our series the constant presence of the intrarhinal sulcus, albeit it was sometimes shallow and not clearly noticeable unless one is aware of the ventral limit of the GA. The presence or absence equally on both sides of the brain suggests that there is no interhemispheric asymmetry (Hanke, 1997). We also found a great hemispheric symmetry of the intrarhinal sulcus in our series of cases. The depth of the intrarhinal sulcus has been associated with the degree of “brain swelling” (Heinsen et al., 1996). While we do not have specific data about brain swelling in the neuropathological report of our cases, it seems unlikely that this condition of the brain is responsible for the appearance of the intrarhinal sulcus.

Other interpretations of the EC sulci in the literature have been proposed (Duvernoy, 2005; Ding and Van Hoesen, 2015), in particular as an imprint of the free edge of the cerebellar tentorium. However, such imprint would probably interfere with the vascular supply of the EC. This fact, plus the almost constant presence of the intrarhinal sulcus, lead us to conclude that it is a sulcus that forms the ventral boundary of the GA, and thence of the EC subfield E_MI_, rather than an imprint of the cerebellar tentorium on the EC surface. The name of intrarhinal sulcus is justified as it lies entirely within the extent of the EC.

### The GA Is Cytoarchitectonically EC Subfield E_MI_

Our study shows that the GA is an EC subfield (E_MI_) which, very likely, coincides with BA34 (Brodmann, 1909). Brodmann named area 34 as “dorsal EC,” thus acknowledging that the EC and area 34 share common features. Although Brodmann does not provide a full cytoarchitectonic description of neither area 34 nor area 28 in his 1909 book, he depicts both area 34 and area 28 in great topographical detail, and specifically he states that both areas are separated by the “inferior rhinal sulcus of Retzius,” which corresponds to the intrarhinal sulcus. It is unclear why Brodmann separates area 34 as distinct of area 28, at the same time that he calls it “dorsal EC,” instead simply EC. It could be speculated that, taking into consideration a similar lamination between subfield E_MI_ and the nonhuman primate EC, Brodmann identified area 34 as a distinct area bases on the similitude with the nonhuman primate EC^7^.

### MRI Identification of the Rostromedial Part of the EC

The MRI scans used for clinical or experimental studies do not allow the segmentation of specific subfields of the EC. For example, subfield E_O_ is very difficult to identify in common MRI images (Insausti et al., 1998b; Pruessner et al., 2002; Wolk et al., 2017). However, given that the GA can be identified in MRI examinations, and the very good (almost perfect) match between subfield E_MI_ and the GA, E_MI_ could be the first EC subfield identifiable in MRI scans.

### Anatomical and Functional Significance

Subfield E_MI_ presents histo- and immunohistochemical peculiarities, that singles it out of other EC subfields. The distribution of parvalbumin-stained neurons is reduced relative to more lateral parts at similar rostro-caudal levels (Braak et al., 1991; Tuñon et al., 1992; Schmidt et al., 1993; Solodkin and Van Hoesen, 1996; Mikkonen et al., 1997). Calbindin and calretinin immunoreactivity stains more heavily the medial part of the EC (Tuñon et al., 1992; Mikkonen et al., 1997), and therefore, it is largely complementary to that of parvalbumin.

Other neurochemical substances in the EC also distinguish rostromedial EC subfields E_MI_ and E_O_. E_MI_ and adjacent parts of E_O_ present a paucity of tyrosine hydroxylase-immunoreactive fibers relative to more lateral parts of the EC (Akil and Lewis, 1994). The increasingly and gradual decrease of tyrosine hydroxylase fiber density make subfield E_MI_ distinguishable from the adjacent E_I_, although the seamless continuation of the layers with subfield E_MI_ support the contention of its being a subfield of EC. The study of the distribution of choline acetyltransferase in the HF shows that E_MI_ displays low density of choline acetyltransferase fibers, density that increases in E_I_ with perfect continuation of the EC layers. Significantly, the boundary seems to be coincident with the intrarhinal sulcus (De Lacalle et al., 1994).

Experimental studies in nonhuman primates reveal that the organization of the projections between the EC and the dentate gyrus of the hippocampus share a similar pattern in the nonhuman primate (Witter et al., 1989) and rodent HF (Ruth et al., 1982). The anterior portion of the hippocampus (ventral part in the rodent), which in humans corresponds to the hippocampal head, receives innervation from rostromedial portions of the EC. The body and tail of the hippocampus (septal or dorsal part in the rodent) are innervated from progressively more lateral and caudal levels of the EC. Cytoarchitectonic studies of the EC in humans show that rostromedial portions of the EC belong to the E_O_ and E_MI_ subfields of the EC. The likely homology between EC subfield E_O_ and the nonhuman primate EC subfield E_O_ (Amaral et al., 1987; Insausti et al., 2002) strongly suggests that this subfield would innervate the head of the hippocampus. Likewise, EC subfield E_MI_, which is not present in the nonhuman primate (it is the only EC cytoarchitectonic subfield that is present exclusively in humans), would also likely innervate the head of the hippocampus (Insausti, 1993).

Up to date, *ex vivo* MRI scans and histology offer the best existing correlation between MRI and the extent of the EC (Adler et al., 2018). The correlation between *ex-vivo* MRI scans of the MTL with the subsequent histological confirmation are useful for localization of MTL structures (Insausti et al., 1998b; Franko et al., 2014; Delgado-Gonzalez et al., 2015; Adler et al., 2018). However, no specific studies on the GA are available. Our results in a small set of cases suggest that the identification of E_MI_ is feasible, and it may be of use in MRI volumetric determinations.

The MRI identification of the GA has functional implications, as topographical differences in the longitudinal axis of the hippocampus are associated with different functional properties (Maguire et al., 2000; Fanselow and Dong, 2010). The identification of an outstanding GA in most subjects would be useful in the determination of EC subfield E_MI_ and partially of E_O_, with the subsequent implications in the volumetric measurements and cortical thickness studies, as, for instance, in Alzheimer disease (Wolk et al., 2017).

### Summary of Findings and Conclusions

The present study characterizes the location of GA and the type of cortex that overlies it. It thus seems to be justified to conclude that the cortex lining the GA is the EC subfield E_MI_, and therefore part of EC. Likewise, BA34 would be identical to EC subfield E_MI_. The topographical situation of the GA in the MTL, plus the coincidence with the EC subfield E_MI_ brings the opportunity of its accurate determination in MRI explorations.

## Ethics Statement

Ethical Committee on Clinical Research of the University Hospital of Albacete, on its meeting of January 2015. Brains obtained before 2002 did not require written consent of next to kin. Brains obtained after this date were obtained under the body and brain donor program of the School of Medicine of the University of Castilla–La Mancha.

## Author Contributions

RI designed the study and wrote the manuscript. MC-P and RI contributed equally to the manuscript. MU and AR carried out the measurements. AI and MM-L performed the cytoarchitectonic analysis of the cases.

## Conflict of Interest Statement

The authors declare that the research was conducted in the absence of any commercial or financial relationships that could be construed as a potential conflict of interest.

## Footnotes

1 Latin nomenclature, from Terminología Neuroanatómica (2017) FIPAT.library.dal.ca. Federative International Programme for Anatomical Terminology is mentioned in *italics* in the text, alongside the common English terms used in neuroanatomical literature (illustrated version in ten Donkelaar et al., 2018).

2 Usually cited in its Latin form, thereby we will continue using this form in the manuscript.

3 The name perirhinal cortex is employed as the combination of BA perirhinalis (BA35) and area ectorhinalis (BA36).

4 The name posterior parahippocampal cortex refers to the posterior part of the PHG, caudal to both entorhinal and perirhinal cortices. This cortical strip was included as part of perirhinal cortex by Brodmann, although it was identified as areas TH and TF by von Economo and Koskinas (1925), as well as in Bailey and von Bonin (1951).

5 Measurements of the intrarhinal sulcus were taken from MRI series of images in the coronal plane of both sexes. Distances encompassed from the first slice in which the intrarhinal sulcus appears, as far as the first slice where the hippocampal fissure could be noticed.

6 The term *verrucae hippocampi* is used as in Klingler (1948) and Simic et al. (2005) to indicate the elevations in the pial surface of otherwise smooth surface of the cortex.

7 Brodmann studied first the cortical parcellation in the nonhuman primate brain, and thereafter in the human brain (Lorente de Nó, personal communication), therefore he was familiarized with the nonhuman primate EC.

## Abbreviations

BA: Brodmann area
BA35: Brodmann area 35 or perirhinal cortex
BA36: Brodmann area 36 or entorhinal cortex
EC: entorhinal cortex
E_C_: caudal subfield of the entorhinal cortex
E_CL_: caudal limiting subfield of the entorhinal cortex
E_I_: intermediate subfield of the entorhinal cortex
E_LC_: lateral caudal subfield of the entorhinal cortex
E_LR_: lateral rostral subfield of the entorhinal cortex
E_MI_: medial intermediate subfield of the entorhinal cortex
E_O_: olfactory subfield of the entorhinal cortex
E_R_: rostral subfield of the entorhinal cortex
GA: *Gyrus ambiens*
GS: *Gyrus semilunaris*
GU: *Gyrus uncinatus*
MTL: medial temporal lobe
PHG: parahippocampal gyrus
